# The study of biomechanics and finite element analysis on a novel plate for tibial plateau fractures via anterolateral supra-fibular-head approach

**DOI:** 10.1038/s41598-023-40842-x

**Published:** 2023-08-19

**Authors:** Yao Lu, HuanAn Bai, Qian Wang, Cheng Ren, Ming Li, Zhong Li, Kun Zhang, Qiang Huang, Teng Ma

**Affiliations:** Department of Orthopedics, Hong Hui Hospital, Xi’an Jiaotong University, Xi’an, 710054 Shaanxi China

**Keywords:** Biomedical engineering, Trauma

## Abstract

For Schatzker type II split-depressed tibial plateau fractures involving the fractures of anterolateral and posterolateral columns (APC), the optimal fixation scheme is controversial. The objectives of this study were: (1) to introduce a newly designed plate for treating APC fractures via biomechanical tests and finite element analysis (FEA), and (2) to compare it with two conventional fixation methods. APC fracture models were created and randomly assigned to three groups (Groups A-C). Group A was fixed with a 3.5-mm lateral locking plate, Group B was fixed with a 3.5-mm lateral locking plate and two 3.5-mm cannulated screws (hybrid fixation). Group C was fixed with the newly designed plate. It is an arched locking plate for fixing the lateral tibial plateau via the anterolateral supra-fibular-head approach. Each fracture model experienced a gradually increasing axial compressive load ranging from 250 to 750 N using a customized indenter. Biomechanical analysis demonstrated that the newly designed plate showed the minimum displacement among the three methods, followed by the hybrid fixation method. Conversely, the 3.5-mm lateral locking plate displayed the maximum displacement in APC fractures (*p* < 0.05). FEA results indicated that at 750 N, the maximum displacements for Groups A-C were measured as 3.06 mm, 2.74 mm, and 2.08 mm, respectively. Moreover, the maximum stresses recorded for the implant in Groups A-C at 750 N were 208.32 MPa, 299.59 MPa, and 143.26 MPa, while for the bone, they were 47.12 MPa, 74.36 MPa, and 40.01 MPa. The overall trends at 250 N and 500 N were consistent with those observed at 750 N. In conclusion, due to good biomechanical performance and FEA results, the newly designed plate represents a promising choice for managing APC fractures of the tibial plateau.

## Introduction

Tibial plateau fracture is one of the most common fractures in knee trauma, accounting for 1% of all fractures^1^. Anatomical reduction and rigid fixation are the first choice for treating such injuries. However, poor reduction and fixation may lead to traumatic arthritis and movement dysfunction. Due to the special geometry and biomechanics of the knee joint, approximately 60% of tibial plateau fractures occur in the lateral column^2,3^. Analysis of the morphological characteristics of the fractures apparent from computed tomography (CT) scan allows for the classification of Schatzker type II fractures into three sub-types: anterolateral single-column (AC) fractures; posterolateral single-column (PC) fractures; and both the anterolateral and posterolateral column (APC) fractures^4^. Sun et al. reported that the proportion of single PC fractures was approximately 15.0% (28/187), and the incidence of fractures involving APC was up to 23.4% (123/525)^4^. These findings indicate that the likelihood of APC fracture should not be neglected when managing Schatzker type II tibial plateau fractures.

Several scholars have proposed that in comminuted tibial plateau fractures, including anterolateral and posterolateral column fragments, the posterolateral fragment should also be properly fixed^4,5^. Currently, there is no consensus on the approaches and implants to treat APC fractures. Several experts have proposed dual-plate fixation using a combined anterolateral and posteromedial inverted L-shaped approach, with the patient in a floating position^4^. Among 41 patients treated with this approach, three patients experienced incision dehiscence or necrosis, and two patients suffered iatrogenic nerve injuries. While the dual-plates provided rigid fixation, it was challenging to directly expose the posterolateral fracture fragment via the posteromedial incision, particularly in patients with well-developed calf muscles or obesity. Even when the posterolateral fragment could be exposed, the operation often resulted in the separation of the popliteal vessels and tibial nerve, increasing the risk of vascular and nerve traction injuries. Zhang et al.^6^ introduced another combined approach for APC fractures, consisting of a conventional anterolateral approach and an inverted L-shaped posterolateral approach. However, two out of seventeen patients treated with this approach experienced aseptic fat liquefaction after the operation. While this approach allowed for direct exposure of the posterolateral fragment, it inevitably caused some iatrogenic injuries at the posterolateral corner of the knee joint. Additionally, the skin bridge between the two incisions had a tendency to develop ischemic necrosis. Zhu et al. used a barrel hoop plate combined with a traditional lateral locking plate to fix APC fractures via a modified Frosch approach^7^. However, in this method, a conventional 2.7 radius T-plate needed to be cut and contoured to obtain the barrel hoop plate. Other scholars have used lateral locking plates in addition to antero-posterior lag screw techniques for managing APC fractures^8^, but further research is needed to verify the effectiveness of this fixation method.

**Figure 1:**
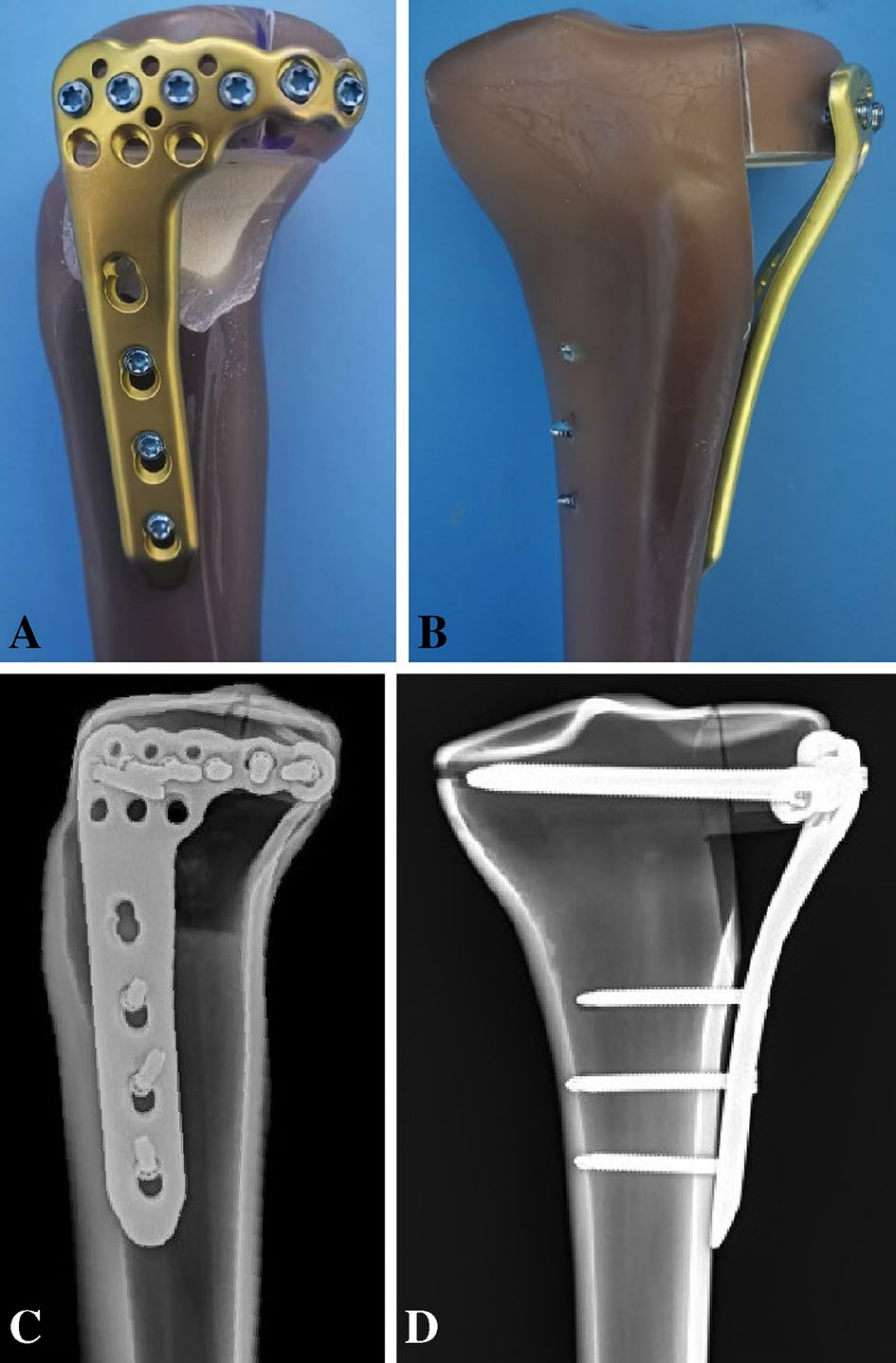
Mock-ups of the newly designed plate in synthetic bones. (**A**) Lateral view of the newly designed plate. (**B**) Anteroposterior view of the newly designed plate. (**C**) Lateral view of the X-ray image. (**D**) Anteroposterior view of the X-ray image.

In this study, our team designed a novel locking anatomic plate (Fig. 1) specifically for APC fractures of the tibial plateau. We aimed to test the biomechanical performance of the newly designed plate for managing such fractures. Additionally, we conducted finite element analysis to explore the effectiveness of this plate. Our hypothesis was that the newly designed plate would provide adequate stability and exhibit superior biomechanical strength and finite element analysis performance.

## Materials and methods

The institutional review board (IRB) approval has been obtained from the ethics committee of Xi’an Hong Hui hospital. A written informed consent was gotten from the volunteer. All methods were carried out based on relevant guidelines and regulations. Thirty synthetic left tibias (type 3401; Sawbones AG, Vashon Island, WA, USA) were used to make APC fracture models. The newly designed plates, 3.5-mm cannulated screws and lateral locking plates (titanium alloy, Naton Medical Instrument Co., Ltd., Tianjin, China) were used to fix the fracture models. The Electroforce 3520-AT electronic universal material testing machine (TA Instruments, New Castle, DE, USA) was used to identify the biomechanical performance of APC fracture models. Vertical displacement was tested by a laser displacement sensor (LN-030-N, Shenzhen Leraun Technology Co., Ltd, China) with a precision of 0.01 mm.

### Fracture models and groups

**Figure 2:**
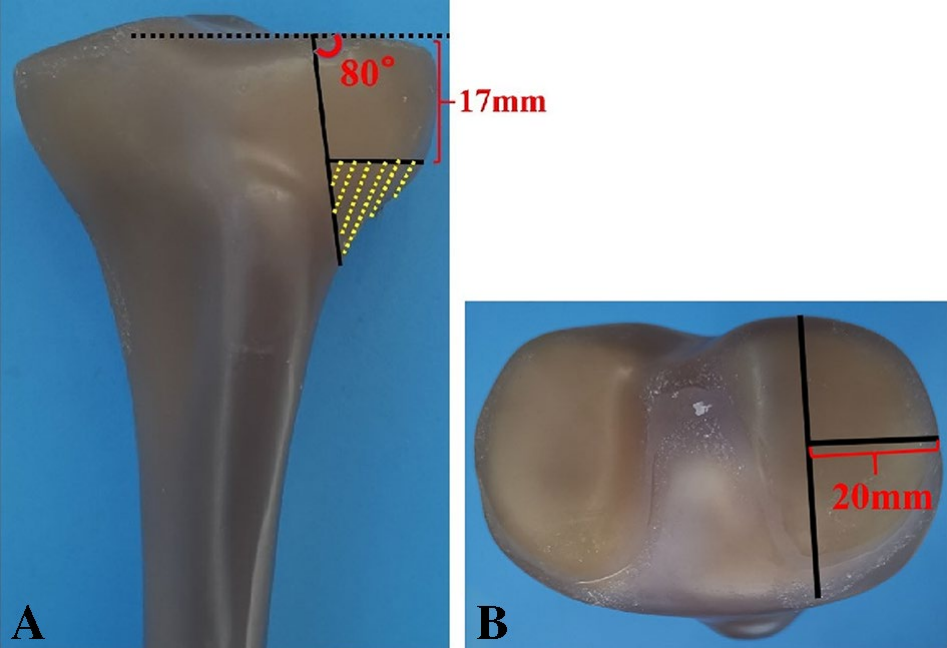
Model of a Schatzker type II split-depressed tibial plateau fracture involving the anterolateral and posterolateral columns.

According to previous studies and our design for APC fractures, thirty synthetic tibias were used to construct APC fracture models. Osteotomy was performed by a thin blade saw. As shown in Fig. 2, the widest part of the lateral tibial plateau fragment was 20 mm. Parallel to the coronal plane, the lateral fragment was divided into two similar-sized parts, generating the anterolateral and posterolateral fracture fragments. The height of the APC fragments was 17 mm. To simulate the effects of fracture depression and subsequent reduction, the yellow shaded section in Fig. 2 was removed, resulting in a simplified APC fracture model. These models were randomly divided into three groups, with ten in each group.

**Figure 3:**
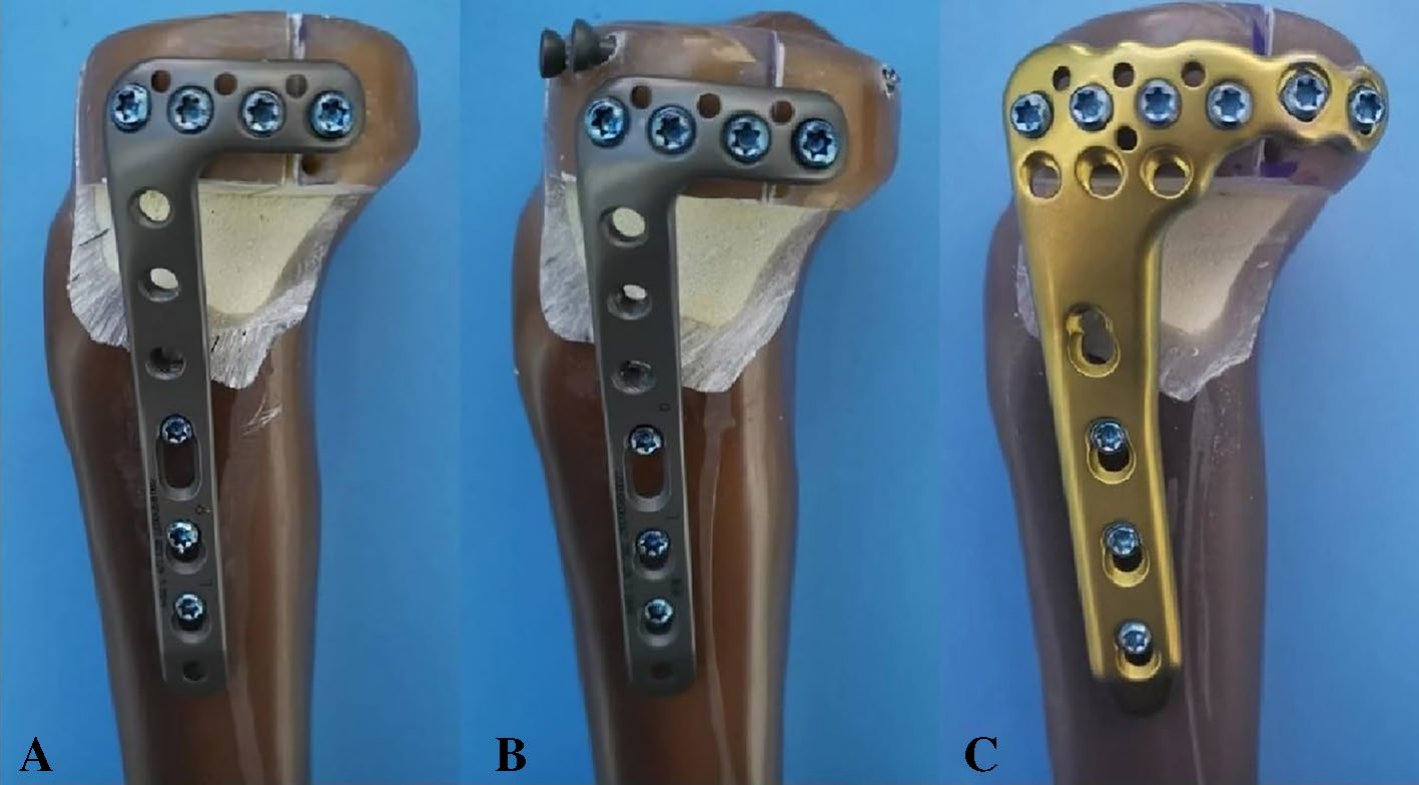
APC fracture models with three different fixation methods in biomechanical tests. (**A**) Fixation of the 3.5-mm lateral locking plate in the APC fracture. (**B**) Fixation of a 3.5-mm lateral locking plate and two 3.5-mm cannulated screws in the APC fracture. (**C**) Fixation of the newly designed plate in the APC fracture. APC stands for Schatzker type II split-depressed tibial plateau fracture involving the anterolateral and posterolateral columns.

As shown in Fig. 3A, group A was fixed with a 3.5-mm lateral locking plate. Four locking screws were inserted parallel to the articular surface, followed by the insertion of three consecutive locking screws in the distal screw holes. Group B was fixed with a 3.5-mm lateral locking plate along with two antero-posterior 3.5-mm cannulated screws (Fig. 3B). The 3.5-mm lateral locking plate was inserted on the lateral surface, positioned 2 mm below the cannulated screws. Figure 3C showed the newly designed plate. This plate was used to fix the anterolateral and posterolateral fracture fragments via the anterolateral supra-fibular-head approach. The horizontal arm of the plate featured six holes, including four locking holes and two universal holes. The screws could wrap around the lateral tibial plateau in an arc shape. Six proximal locking screws were inserted parallel to the articular surface. The universal holes were used to properly fix the posterolateral fragment through the space above the fibular head. Previous literature have demonstrated that the space between the apex of the fibular head and the lateral wall of the plateau is sufficient for the horizontal arm of the plate to pass through^9^. The same operator reduced and fixed these fracture models according to the standard procedures.

### Biomechanical testing

Each assembled synthetic tibial model was fixed vertically in the biomechanical equipment. A vertical load was applied to the center of the lateral tibial plateau by a custom cylindrical indenter with a diameter of 30 mm. Previous studies have shown that under normal gait, the biomechanical load on the knee joint is approximately two to three times of the body weight^10^. The lateral tibial plateau bears about 45% of this load, while the medial tibial plateau bears about 55%^11^. To simulate the loads acting on the lateral plateau, axial peak loads of 250 N, 500 N, and 750 N were set for the synthetic fracture models. These load values corresponded to approximately one to three times of the body weight borne by the lateral tibial plateau of adults (weighing 60 kg). The biomechanical equipment simulated the static phase for the three fixation methods mentioned above. After assembling the APC fracture model on the equipment, gradually increasing axial loads were applied to the model at a load speed of 10 N/s. The vertical displacement was measured by the laser displacement sensor. The displacement values were recorded by a specific software. A load–displacement curve was generated for each fracture model. Additionally, failure was defined as the point where the vertical displacement of the APC fragments was 3 mm^12^. The maximum peak loads were set at 750 N or at the loads corresponding to a displacement of 3 mm for the APC fragments. The vertical displacements at three stated loads and failure loads were used to assess the effectiveness of the three implants.

### FEA performance

A healthy male volunteer with no history of knee joint or systemic diseases was recruited. A CT scan was performed on the knee to ankle region using a 64-row spiral CT machine, with a slice thickness of 0.625 mm. The CT images were stored as a DICOM format file into the interactive medical image control system (Mimics 19.0, Materialise, Leuven, Belgium). Based on the gray values of the tissues and segmentation of regions, a 3D reconstruction model of the tibia was generated. The 3D images were then divided into surface meshes by Mimics’ Magics 9.9 rigid-lattice division program. Subsequently, the FEA modules of Mimics software were used to optimize these 3D models and create surface mesh models. These surface mesh models were further converted into volume mesh models using the Mesh tool (ANSYS, Inc., Canonsburg, PA, USA). The volume mesh models were imported back into Mimics to assign material performances. A convergence study was conducted using 10-node quadratic tetrahedral elements and 4-node linear elements. The maximum Degree of Freedom was checked for various variables, such as strain energy and displacement, ensuring that they were within 5% for both types of elements, and no maximum stress points were identified.

**Figure 4:**
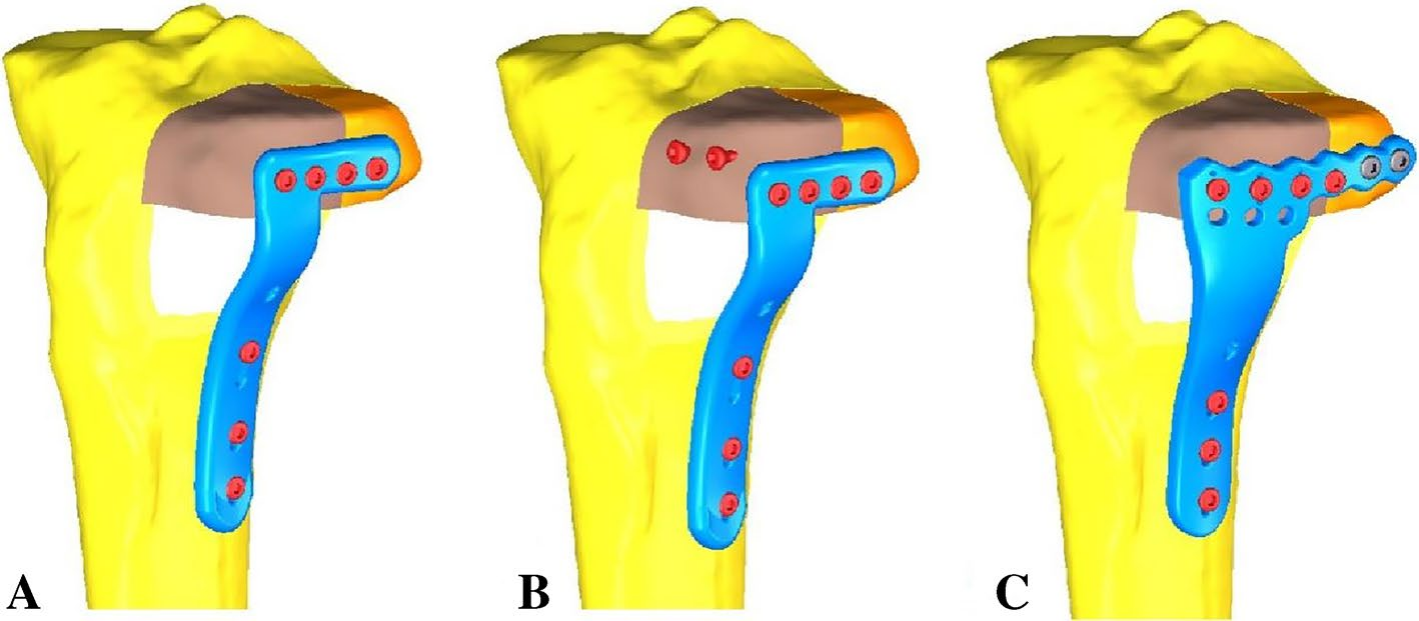
Three different internal fixation after assembly of the finite element model. (**A**) Fixation of the 3.5-mm lateral locking plate in APC fracture. (**B**) Fixation of a 3.5-mm lateral locking plate and two 3.5-mm cannulated screws in the APC fracture. (**C**) Fixation of the newly designed plate in the APC fracture. APC stands for Schatzker type II split-depressed tibial plateau fracture involving the anterolateral and posterolateral columns.

The Young's modulus for cortical bones was set as 14,000 MPa, for cancellous bones as 700 MPa, and for plates and screws as 110,000 MPa. The Poisson's ratio for these materials was set at 0.3, based on previous literature^13,14^. All plates and screws used in the study were made of titanium alloy. The 3D models of these plates and screws were created by a computer-aided design software, according to specific information provided by the manufacturer. Frictional contacts were defined for all contact situations. A friction coefficient of 0.4 was selected based on the literature^15^. The fracture lines were cut to obtain the APC fracture models (Fig. 4). Different implants were assembled onto the fracture models. These models were divided into three groups and fixed by the 3.5-mm lateral locking plate, hybrid fixation devices, or the newly designed plate, respectively. Table 1 displayed the numbers of elements and nodes for the three fixation models.

**Table 1:** Number of nodes and elements for the three different models.

Model	Nodes	Elements
Group A	373,876	1,992,716
Group B	446,625	2,325,857
Group C	382,510	2,032,605

Group A: the 3.5-mm lateral locking plate. Group B: a 3.5-mm lateral locking plate plus two 3.5-mm cannulated screws. Group C: the newly designed plate.

The inferior of the tibia shaft was fixed in all degrees of freedom. The APC fragments were compressed by the axial loads of 250 N, 500 N, and 750 N. All fracture models were analyzed by the software ANSYS Mechanical APDL 19.0 (ANSYS, Inc., USA). The FEA was performed to simulate a static experiment for the three fixation methods. The maximum vertical displacement of each model was analyzed. Additionally, the von Mises stress distribution and the maximum von Mises stress of each model were also tested.

### Statistical Analysis

One-way analysis of variance was taken on the data to identify whether vertical displacement of fragments and failure loads differed in the three fixation methods. Fisher’s post hoc test and least-significant difference (LSD) criterion were applied for multiple group comparisons. *p* < 0.05 was used to indicate statistical significance. Data were analyzed using SPSS 22.0 (SPSS, Inc., Chicago, IL, United States).

## Results

### Biomechanical performance of three groups

The vertical displacements of the three groups under axial loads of 250 N, 500 N, and 750 N are presented in Table 2. There is a clear hierarchy of displacement among the fragments in the APC fracture models at these load levels: the newly designed plate (Group C) exhibited the least displacement, followed by the hybrid fixation group (Group B), while the 3.5-mm lateral locking plate (Group A) showed the highest displacement. Statistically significant differences were observed among the three groups (*p* < 0.05).

**Table 2:** Vertical displacement of APC fractures at three different load levels, load to failure.

Group	Vertical displacement (mm)			Load to failure (N)
	250 N	500 N	750 N	
A	1.35 ± 0.10	2.33 ± 0.18	3.29 ± 0.30	628.50 ± 33.51
B	1.12 ± 0.11	1.84 ± 0.15	2.99 ± 0.23	759.84 ± 30.87
C	0.84 ± 0.04	1.58 ± 0.17	2.36 ± 0.24	911.52 ± 27.93
P (A-B)^a^	0.001	0.001	0.02	0.001
P (B-C)^a^	0.001	0.002	0.001	0.001
P (C-A)^a^	0.001	0.001	0.001	0.001

^a^Significant difference.

The failure loads for each fracture model are also summarized in Table 2. Group C demonstrated the highest failure loads, with values significantly higher than those of the other two fixation methods (*p* < 0.05). The hybrid fixation group (Group B) was capable of bearing more loads than Group A. The failure loads for Group A were 628.50 ± 33.51 N, for Group B were 759.84 ± 30.87 N, and for Group C were 911.52 ± 27.93 N. These findings indicate that the newly designed plate possesses superior biomechanical properties compared to the 3.5-mm lateral locking plate and the hybrid fixation implants in terms of vertical displacement and failure loads.

### Finite element analysis of three groups

**Figure 5:**
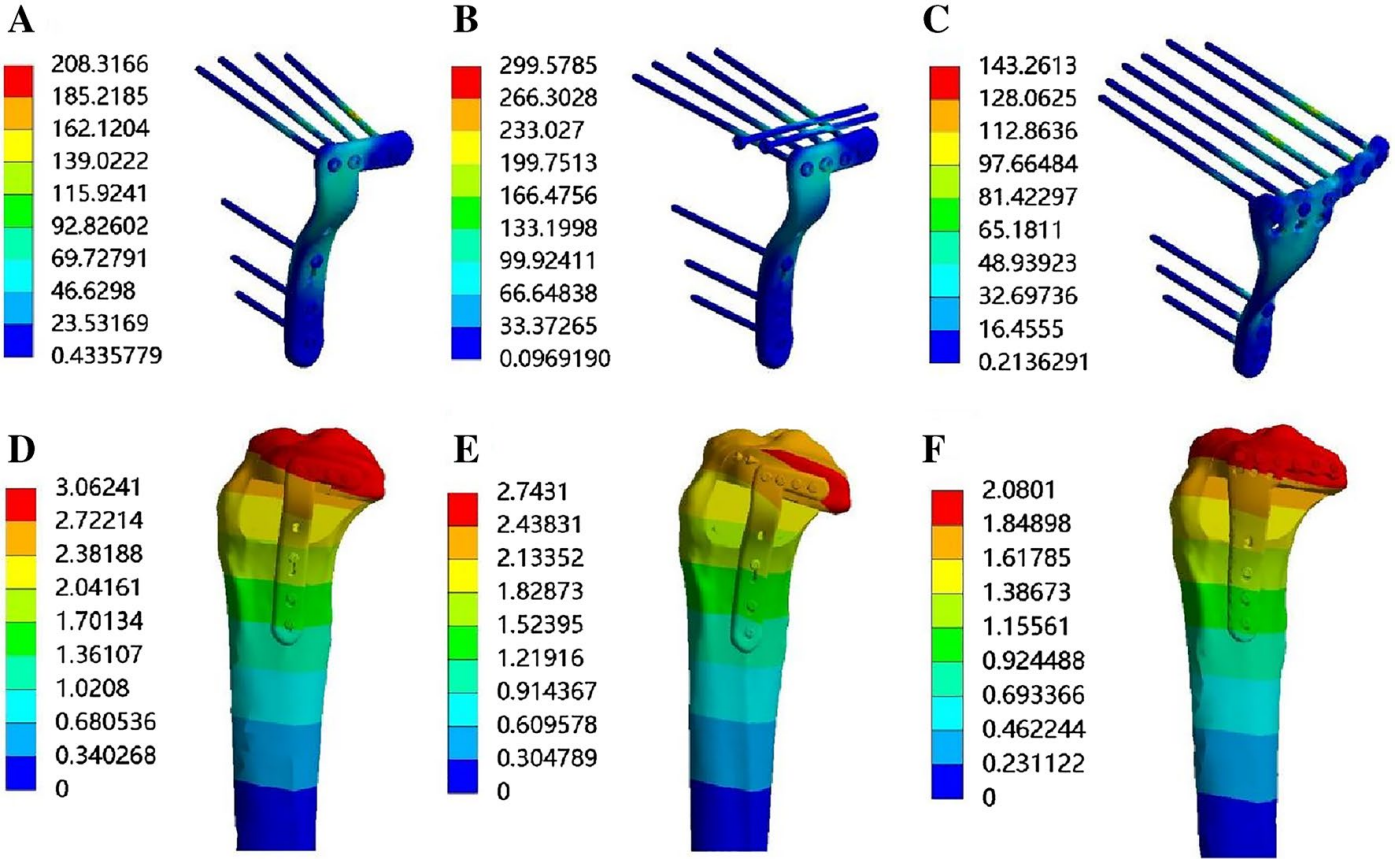
Stress distribution diagram and displacement field of the three finite element models. (**A**) Stress distribution of model A in APC fracture. (**B**) Stress distribution of model B in APC fracture. (**C**) Stress distribution of model C in APC fracture. (**D**) Displacement field of model A in APC fracture. (**E**) Displacement field of model B in APC fracture. (**F**) Displacement field of model C in APC fracture. APC stands for Schatzker type II split-depressed tibial plateau fracture involving the anterolateral and posterolateral columns.

Table 3 presents the maximum displacements of the finite element models for APC fractures. Under axial loads of 750 N, the maximum displacements for Groups A, B, and C were 3.06 mm, 2.74 mm, and 2.08 mm, respectively (Fig. 5D,E,F). The displacement trends from 250 to 750 N were consistent, with the displacements of the APC fragments in each fixation model gradually increasing as the axial loads increased.

**Table 3:** Maximum displacement of the finite element models of APC fractures.

Group	Max displacement (mm)		
	250 N	500 N	750 N
A	1.03	2.05	3.06
B	0.93	1.84	2.74
C	0.69	1.39	2.08

The von Mises stress distributions for the 3.5-mm lateral locking plate (Group A) mainly concentrated on the two locking screws of the horizontal arm in contact with the coronal fracture line and the corner of the L-shaped plate (Fig. 5A). For the hybrid fixation implants (Group B), the von Mises stress distributions focused on the corner of the L-shaped plate and the midsection of the outer cannulated screw (Fig. 5B). In the newly designed plate (Group C), the von Mises stress distribution was observed at the midsection of the proximal six screws uniformly and the corner junction of the horizontal and longitudinal arms (Fig. 5C). The von Mises stress increased in all three fixation implants as the axial loads increased. With a load of 750 N applied to the models, the maximum von Mises stress values for Group A, B, and C were 208.32 MPa, 299.59 MPa, and 143.26 MPa, respectively. The von Mises stress distribution followed a similar pattern as the loads increased from 250 to 500 N. The von Mises stress values under the specified loads are displayed in Table 4. Furthermore, as shown in Table 5, the maximum von Mises stress acting on the bone under the load of 750 N was 47.12 MPa, 74.36 MPa, and 40.01 MPa for Group A, B, and C, respectively.

**Table 4:** Maximum von Mises stress of the finite element models of APC fractures in internal fixation method.

Group	Max von Mises stress (MPa)		
	250 N	500 N	750 N
A	71.90	140.73	208.32
B	61.60	152.13	299.59
C	49.22	96.71	143.26

**Table 5:** Maximum von Mises stress of the finite element models of APC fractures in bone.

Group	Max von Mises stress (MPa)		
	250 N	500 N	750 N
A	16.10	31.67	47.12
B	15.71	37.01	74.36
C	13.49	26.70	40.01

## Discussion

Several surgeons have successfully applied lateral plate fixation to manage PC fractures via an extended anterolateral or anterolateral supra-fibular-head approach^9,16^. Anterolateral approaches offer the advantages of simple operation and a low risk of damaging important anatomical structures at the posterolateral corner. Based on this, our team suggests that the anterolateral supra-fibular-head approach might be a good choice for Schatzker type II split-depressed tibial plateau fractures involving the anterolateral and posterolateral columns (APC fractures). Currently, experts in the management of APC fractures primarily focus on different approaches, and there have been few reports on specially designed plates for APC fractures. Although the traditional 3.5-mm lateral locking plate can provide good support for single AC or PC fractures, it may not be suitable for APC fractures. Moreover, the available types of fixation devices for both anterolateral and posterolateral column fragments are relatively limited. There is no consensus on the optimal fixation method for APC fractures of the tibial plateau.

In order to improve the challenging situation of treating APC fractures, our team has designed a novel plate via the anterolateral supra-fibular-head approach. The newly designed plate is in an inverted L-shape, with six holes on the horizontal arm forming an arc that encompasses the entire anterolateral and posterolateral columns of the tibial plateau. This plate effectively fixes the posterolateral fragment from above the fibular head. The design concept is similar to the "barrel hoop plate" used in previous studies for PC fractures^7^. However, compared to our newly designed plate, the barrel hoop plate lacks a plate body that extends to the tibia shaft, making it difficult to use alone in clinical practice. Furthermore, the barrel hoop plate is typically created by pre-bending and trimming a thin distal radius plate, which may not provide sufficient support or rigid fixation. Additionally, the horizontal arm of the barrel hoop plate is not long enough. When both the anterolateral and posterolateral columns of the tibial plateau are affected, the barrel hoop plate needs to be combined with other internal fixations, increasing surgical trauma and prolonging operation time. In contrast, the long horizontal arm of our newly designed plate can simultaneously fix the anterolateral and posterolateral columns. The plate is anatomically designed to ideally adhere to the lateral tibial plateau. The rear two holes of the horizontal arm are universal holes, providing angle-stable support and preventing screws from penetrating the cortex.

Biomechanical tests have shown that the newly designed plate exhibits better strength for APC fractures compared to the 3.5-mm lateral locking plate and hybrid fixation implants. The FEA results also indicate that the newly designed plate group experiences the least displacement for APC fragments, which aligns with the biomechanical findings. Furthermore, by analyzing the maximum von Mises stress and stress distribution using 3D models, our team has determined that the newly designed plate exhibits lower maximum von Mises stress and a more balanced stress distribution compared to the 3.5-mm lateral locking plate and hybrid fixation implants. These findings suggest that the newly designed implant carries little biomechanical failure risk and is a reasonable design.

There are several limitations of this research. While cadaveric bones are considered the optimal test material, our team used synthetic tibias instead. However, synthetic bones offer certain advantages over cadaveric bones, such as standardized dimensions and properties for each specimen. Additionally, this study did not take into account the factors that affect knee stress and stability, including ligaments, muscles, and other soft tissues. The biomechanical tests conducted in this research were relatively simple. Clinical work reveals diverse morphologies of APC fractures, which our fracture model cannot fully simulate. Furthermore, the applicability of the newly designed plate to different APC fracture models has not been verified. Further design of APC fracture models will be necessary. Lastly, the clinical application of the newly designed plate for APC fractures of the tibial plateau requires further verification.

## Conclusion

The newly designed plate, inserted via the anterolateral supra-fibular-head approach, demonstrates excellent biomechanical performance and FEA results, making it a promising choice for managing APC fractures. This surgical technique has a low risk of iatrogenic injury to important anatomical structures in the region. Consequently, it has great potential for widespread clinical application in treating APC fractures of the tibial plateau.

## Data Availability

The datasets analyzed during the current study are available from the corresponding author upon reasonable request.
